# A systematic review and meta-analysis of the prevalence of caregiver acceptance of malaria vaccine for under-five children in low-income and middle-income countries (LMICs)

**DOI:** 10.1371/journal.pone.0278224

**Published:** 2022-12-01

**Authors:** Sahabi Kabir Sulaiman, Muhammad Sale Musa, Fatimah Ismail Tsiga-Ahmed, Farouq Muhammad Dayyab, Abdulwahab Kabir Sulaiman, Abdulaziz Tijjani Bako

**Affiliations:** 1 Department of Medicine, Yobe State University Teaching Hospital, Damaturu, Nigeria; 2 Department of Community Medicine, Bayero University Kano, Kano, Nigeria; 3 Aminu Kano Teaching Hospital, Kano, Nigeria; 4 Infectious Diseases Hospital, Kano, Nigeria; 5 Department of Medicine, Kwanar Dawaki Isolation Center Kano, Kano, Kano State, Nigeria; 6 Department of Medicine, King Hamad University Hospital, Muharraq, Bahrain; 7 Murtala Muhammad Specialist Hospital, Kano, Nigeria; 8 Center for Outcomes Research, Houston Methodist, Houston, Texas, United States of America; The Technical University of Kenya, KENYA

## Abstract

**Introduction:**

Malaria is the second leading cause of death in children after diarrheal disease, with low- and middle-income countries (LMICs) accounting for over 9 in 10 incidence and deaths. Widespread acceptance and uptake of the RTS,S vaccine, recently approved by the world health organization (WHO), is projected to significantly reduce malaria incidence and deaths. Therefore, we conducted this systematic review and meta-analysis with the aim to determine the malaria vaccine acceptance rate and the factors associated with acceptance.

**Methods:**

We searched six databases including Google Scholar, PubMed, Cochrane, African Index Medicus, The Regional Office for Africa Library, and WHO Institutional Repository for Information Sharing (IRIS) to identify studies evaluating the malaria vaccine acceptance rate. This systematic review and meta-analysis followed the Preferred Reporting Items for Systematic Review and Meta-analysis (PRISMA) guidelines. Studies were included if they were original articles published in the English language in peer-reviewed journals and assessed the prevalence of willingness to accept a free malaria vaccine, and not qualitative. The risk of publication bias was checked using both Beggar’s funnel plot and Egger’s test, while the I^2^ statistic was used to assess the heterogeneity of the included studies. Study quality was determined using the Newcastle-Ottawa scale. A meta-analysis was performed using a random effects model to evaluate the pooled prevalence of malaria vaccine acceptance. The protocol for this article was registered prospectively on the International Prospective Register for Systematic Reviews (PROSPERO), with ID number CRD42022334282).

**Results:**

Our analysis included 11 studies with a total sample size of 14, 666 participants. The aggregate malaria vaccine acceptance rate was 95.3% (95% CI:93.0%–97.2%). Among the general population, the acceptance rate was 96.3% (95% CI:92.0%–99.0%) and among mothers, it was 94.4% (95% CI:90.8%–97.2%). By country, Nigeria had the highest acceptance rate (97.6%, 95% CI:96.0%-98.8%), followed by Ghana (94.6%, 95% CI:93.8%-95.3%) and Tanzania (92.5%, 95% CI:84.4%-97.8%). Sociodemographic determinants of vaccine acceptance included place of residence, tribe, age, sex, occupation, and religion. Reasons for low acceptance included safety concerns, efficacy profile, vaccine’s requirement for multiple injections, and poor level of awareness.

**Conclusion:**

Future efforts should be focused on identifying factors that may improve the actual uptake of the RTS,S vaccine in malaria-endemic communities.

## Introduction

Malaria remains a major public health problem, with almost half of the world’s population at risk of infection, thereby contributing substantially to the global burden of morbidity and mortality [1]. Moreover, the sequel to the disruptions caused by the COVID-19 pandemic, the global incidence, and death rates of malaria have risen to 241 million and 627,000 in the year 2020, respectively, representing an increase of 14 million more cases and 69, 000 more deaths compared to 2019 [2]. Approximately 9 out of 10 global malaria incidence and deaths are accounted for by the WHO African region, with six countries in the region accounting for more than half of the global incidence and deaths [2]. Children under five years of age, particularly those living in the WHO African sub-region and other endemic areas, are the most vulnerable age group, accounting for more than two-thirds of malaria deaths [2]. Malaria imposes substantial costs to both individuals and governments with an estimated direct cost of at least US $12 billion annually [1].

Universal acceptance and uptake of the RTS,S vaccine, a WHO-approved vaccine that offers protection against the P. falciparum malarial parasite, has been envisioned to substantially reduce the global burden of morbidity and mortality due to malaria, particularly among children [3]. However, prior research has shown that acceptance and uptake of childhood vaccines, especially in low- and middle-income countries (LMICs) is suboptimal, with the pooled prevalence of full immunization among children reported to be as low as 1 in 10 in certain parts of Nigeria [4]. Moreover, recent evidence suggests that vaccine “hesitancy”, defined by the WHO as “a delay in the acceptance or refusal of vaccination despite the availability of vaccination services” [5]—albeit a global phenomenon- is particularly burgeoning in LMICs [6].

Since the initial roll-out of the RTS, S vaccine, the studies conducted to assess willingness to accept the RTS,S vaccine among caregivers of under-five children have reported inconsistent estimates of the pooled prevalence of vaccine hesitancy. Therefore, in this study, we sought to perform a systematic literature review and meta-analysis to determine the prevalence of willingness to accept the RTS,S malaria vaccine in LMICs. The findings of this study may guide policymakers and other stakeholders on the selection of appropriate public health measures to maximize vaccine coverage and uptake in malaria-endemic communities.

## Methods

This review was performed in accordance with the Preferred Reporting Items for Systematic Reviews and Meta-Analyses (PRISMA) guidelines [7]. The study protocol was registered in PROSPERO (CRD42022334282).

We conducted our literature search on the 6th of April 2022 in multiple databases, including Google Scholar, PubMed, Cochrane, African Index Medicus, the Regional Office for Africa Library, and the WHO Institutional Repository for Information Sharing (IRIS) to identify published studies assessing malaria vaccine acceptance/hesitancy. A detailed search strategy was developed for PubMed and adapted for the other databases (**S1 Table**). The Boolean operators, ‘AND’ and ‘OR’, and truncation were appropriately utilized in combination with keywords. Thus, the search terms include “malaria” combined with “vaccine” OR “vaccination” OR “immunization” OR “acceptance” OR “uptake” OR “willingness” OR “awareness” OR “perception” for the literature search appropriate for the database. MeSH (Medical Subject Headings) terms for these keywords were also added to expand the search. Search terms were matched with the names of all countries falling under the low- or middle-income classification according to the World Bank definition [8].

The studies included in this analysis are original full-text articles published in peer-reviewed journals, assessing the prevalence of willingness to accept a free malaria vaccine, and published in the English language. Studies were excluded if they were preprints or abstract-only papers, only assessing participants’ willingness to pay for the vaccine, or solely qualitative.

**Fig 1:**
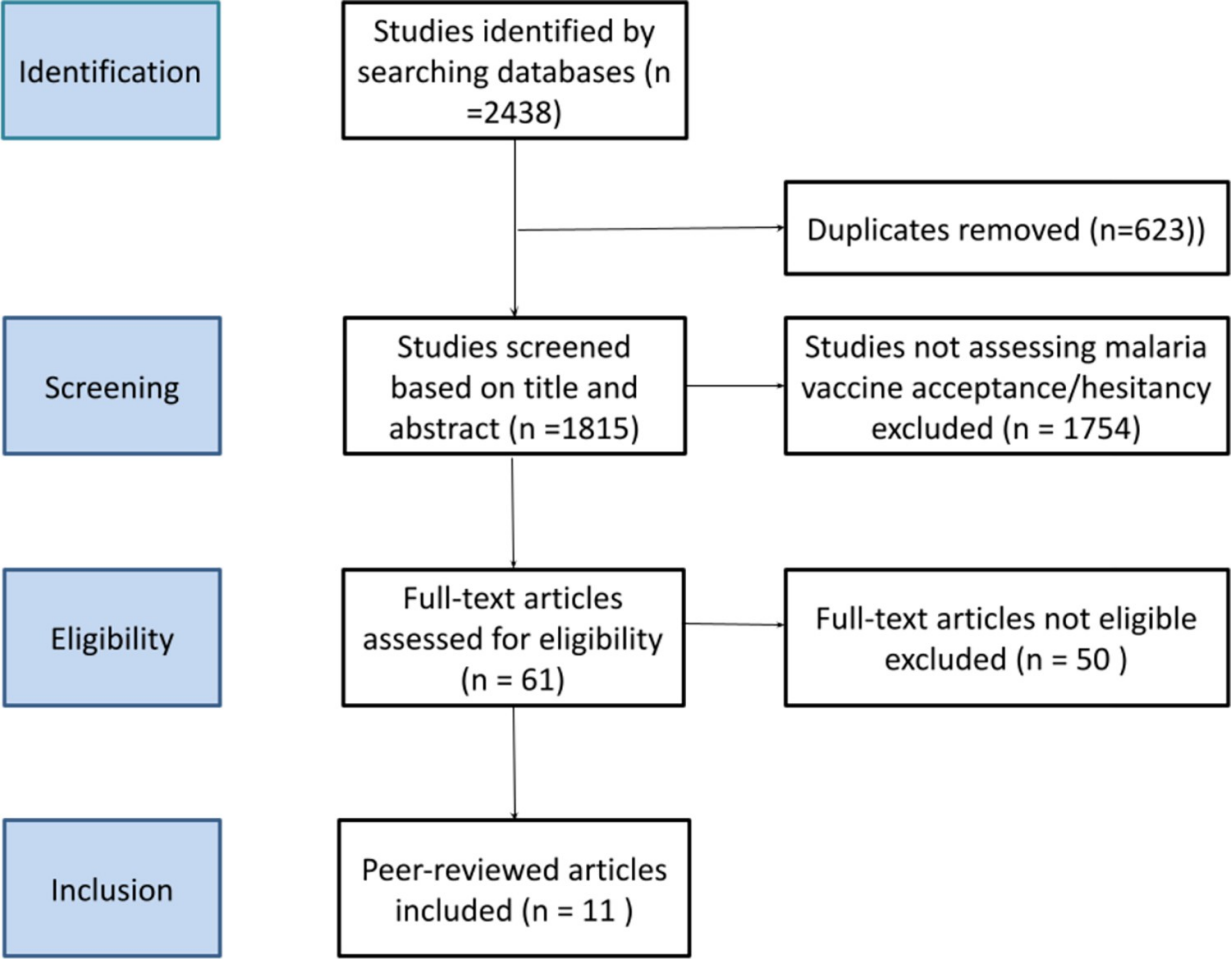
PRISMA flow diagram of literature search.

Following the literature search and screening of abstracts-only and preprint papers, we identified a total of 2438 studies and screened 61 studies based on title and abstract. Of the 61 screened studies, 50 were further excluded based on other inclusion/exclusion criteria, bringing the total number of included studies to 11 (**Fig 1**).

### Data extraction

All the included studies were first entered into Zotero software (version 6.0.15) for the removal of duplicate articles. Thereafter, data were independently extracted from all included articles by one author (SKS) using a standardized data extraction form built in Microsoft Excel and adapted from the Joanna Briggs Institute (JBI) [9] data extraction form. Information extracted from eligible studies included: the name of the first author, the year the study was conducted and published, the country where it was conducted, the sample size of the study, the study design employed, the target population (general public, mothers, women, etc.), percentage of female participants, acceptance rate reported, and factors associated with acceptability (if reported).To further ensure the accuracy of the extracted data, two senior authors (FIT and ATB) independently reviewed and discussed all the data extracted from the included studies.

#### Critical appraisal of included studies (quality assessment)

Two authors (SKS and MSM) independently reviewed all included studies to assess their methodological quality using an adapted version of the Newcastle-Ottawa Scale (NOS) [10]. The scale has seven items divided into three domains: 1) Selection, which has four items and a maximum score of five; 2) Comparability, which has one item and a maximum score of two; and 3) Outcome, which has two items and a maximum score of three. Studies were rated as having low (1–4), moderate (5–7), or high (8–9) quality of evidence.The scores of the two authors were compared, and two senior authors (FIT and ATB) resolved disputed scores by reviewing and discussing the articles and deciding on a final consensus quality score (**S2 Table**).

### Statistical analysis

A pooled prevalence of malaria vaccine acceptance was estimated from all studies utilizing inverse variance weights. All pooled proportions were presented using forest plots. The percentage of total variation (heterogeneity) across studies was evaluated using the I^2^measure. Heterogeneity between the studies was considered mild if I^2^ falls between 0% to 40%, moderate if 30% to 60%, substantial if 50% to 90%, and considerable if 75 to 100% [11]. As recommended [12], we chose the random effects model due to the considerable heterogeneity in the reported acceptance rates across included studies (Heterogeneity chi^2^ = 338.19; p<0.001;I^2^ = 96.75%). We additionally performed subgroup analyses, thereby stratifying the analysis of pooled acceptance rate by study population and country of publication. The Freeman-Tukey double arcsine transformation was enabled to prevent the exclusion of some studies with proportions close to or at 1. We report the pooled proportions and weighted mean differences with their 95% confidence intervals (CI). The meta-analysis was performed using the metaprop command in Stata Version 15IC (StataCorp, College Station, Texas USA) [13]. A p-value of 0.05 was considered significant.

#### Publication bias

**Fig 2:**
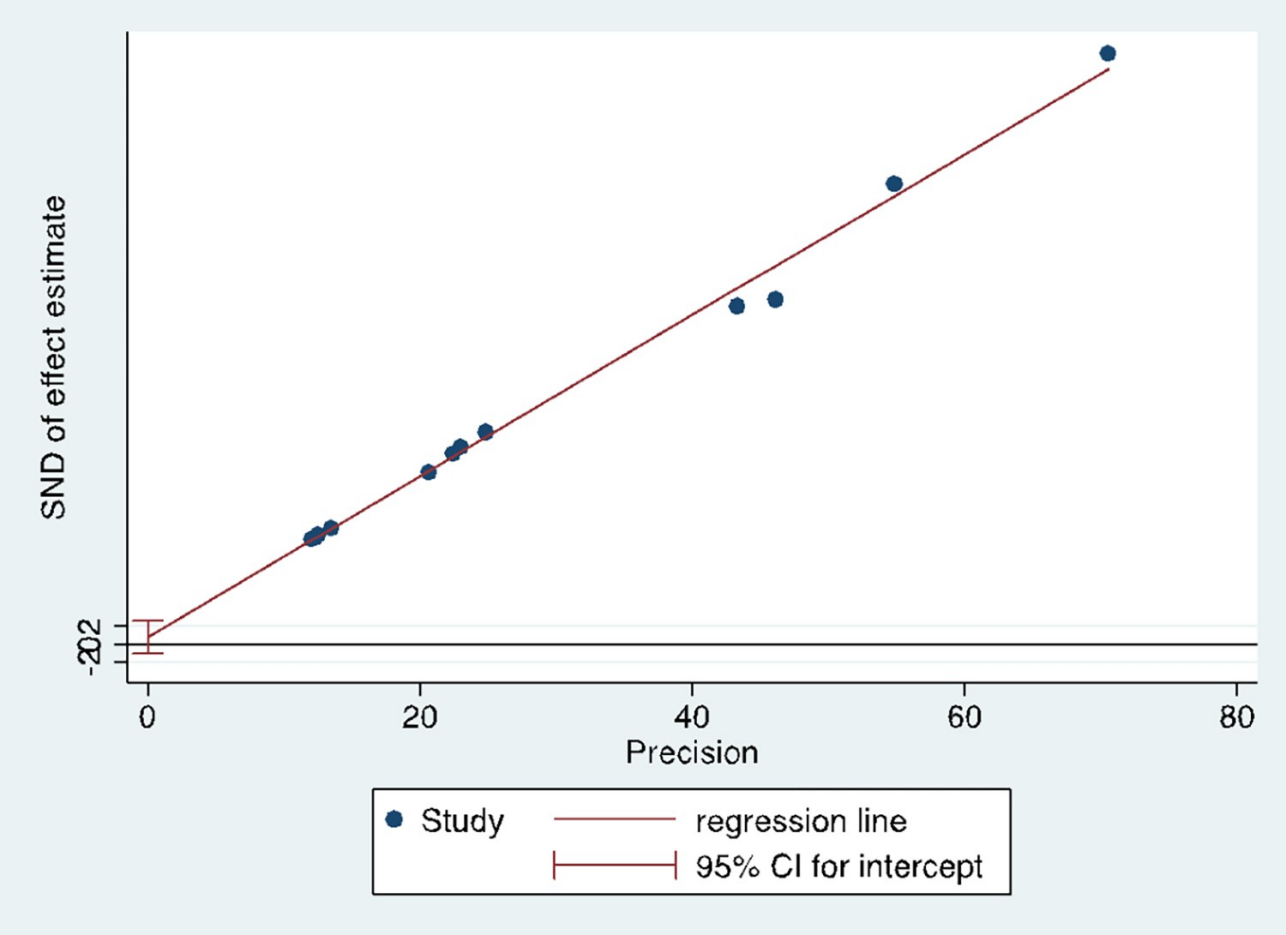
Egger graph to test small study effect.

Publication bias among the included studies was assessed using both Beggar’s funnel plot [14] and Egger’s test [15], with a P > 0.05 indicating no statistically significant evidence of publication bias. Studies included were found to be highly asymmetrical using Beggar’s funnel plot (**S1 Fig**), implying the potential existence of publication bias. However, no evidence of publication bias was demonstrated using Egger’s (weighted regression) test (p = 0.369) (**Fig 2**).

## Results

A total of eleven studies met the inclusion criteria and were analyzed. The details of these studies are provided in **Table 1**.

**Table 1:** Summary of included studies reporting acceptance rates of a malaria vaccine.

Author	Study year: conducted [published]	Country	Sample size (n)	Study design	Target population	Females (%)	Acceptance (n)	Acceptance rate (%)	Factors associated with acceptability	Quality score
Ojakaa et al. [16]	2010 [2014]	Kenya	1870	CS	General	47	1645	88	Region of residence (coast, central, Nyanza, eastern, northeastern, rift valley, western) and higher satisfaction with health services was associated with higher acceptance. Conversely, lower acceptance was observed among caregivers above 50 years	Moderate
Mtenga et al [17]	2013 [2016]	Tanzania	2123	CS QC	Mothers	100	1788	84.2	Being a civil servant, being Christian, belonging to the Hangaza tribe, and residing in the Kagera region are associated with higher acceptance rates	Moderate
Romore et al. [18]	2011 [2015]	Tanzania (Mainland)	4974	CS	Mothers	100	4690	94.3	NR	High
Romore et al. [18]	2011 [2015]	Tanzania (Zanzibar)	528	CS	Mothers	100	511	96.8	NR	High
White et al. [19]	NR [2018]	Peru	143	CS QC	General	62	142	99.3	Acceptability waned with the prospect of multiple injections and presumed vaccine efficacy, respectively, for both adults and children	Moderate
Ughasoro et al. [20]	NR [2018]	Nigeria	155	CS	General	67.9	154	99.3	NR	Fair
Chukwuocha et al. [21]	NR [2018]	Nigeria	500	CS	Mothers	100	481	96.2	Caregiver’s perception was significantly associated with the intent to comply with a prospective malaria vaccine.	Low
McCoy et al. [22]	2019 [2021]	Sierra Leone	615 (CS)	CS QC	General	59.5	592	96.3	A majority would accept if the vaccine is safe and effective	Moderate
Immurana et al. [23]	2019 [2022]	Ghana	3004	CS	Mothers	100	2842	94.6	Belonging into the age group 27 to 38 years; having a female-headed household, and mothers with children aged 5 to 24 months, rise in household size, and mother’s awareness of a malaria vaccine was associated with a higher acceptance. Belonging to the Methodist and Pentecost/Charismatic faiths, residing in the Upper West region, and being from the richest households, a rise in the number of children aged five years or below was associated with lesser odds of willingness to uptake the vaccine	High
Tabiri et al. [24]	2019 [2021]	Ghana	424	CS	General	99.5	399	94.1	Acceptance rate was higher among those having parents with a higher (tertiary) level of education, and parents whose child experienced fever compared to those whose child had abscess after immunization. Conversely, the acceptance rate was lower among parents who thought of vaccines being too many for children.	High
Musa-Booth et al. [25]	2020 [2021]	Nigeria	180	CS	Mothers	100	176	98	NR	High
Onyekachi et al. [26]	NR [2021]	Nigeria	150	CS	General	NR	146	97.3	A majority believe the poor level of awareness, and lack of vaccine availability affect acceptability, and a majority also believed it is not affected by culture	Low

CS, cross-sectional study; QC, qualitative comments; NR, not reported; TBV, transmission-blocking vaccine.

### Characteristics of studies included in this review

Our analysis included 11 studies with a total sample size of 14, 666 participants. A majority (n = 8) [19–26] of the studies were published on or after 2018, while the remaining (n = 3) [16–18] studies were published before 2018. The most recently published study wasin 2022, [23] and the oldest study was published in 2014 [16]. Among all included studies, ten were conducted in Africa, [16–18, 20, 21, 23–26] and one was conducted in South America [19]. Stratified by country, four studies were conducted in Nigeria [20, 21, 25, 26], two were conducted in Ghana [23, 24], one in Kenya [16], one in Sierra Leone [22], two in Tanzania [17, 18] and one in Peru [19].

In terms of sample size, nine of the studies had a sample size between 150 and 3004. Of the remaining two studies, one had the largest sample size (5502) [18] and the other had the smallest sample size (143) [19]. Except for one study, which did not report its study design [26], all other studies were cross-sectional, with three [17, 19, 22] using a mixed-methods approach and the remaining eight studies utilizing quantitative analysis [16, 18, 20, 21, 23–26].

Furthermore, a majority of the studies (n = 6) were conducted among the general population (including male and female caregivers) [16, 19, 20, 22, 24, 26], while the remaining five studies exclusively targeted mothers of under-five children [17, 18, 21, 23, 25]. The highest proportion of female participants reported was 99.5% [24], while the least reported was 47% [16].

### Malaria vaccine acceptance rate

Overall, the reported acceptance rate for the malaria vaccine ranged from 84.2% [17] to 99.3% [19]. Among studies targeting the general population, the acceptance rate ranged from 88% [16] to 99.3% [19]. As for studies having mothers as the target population, the acceptance rate ranged from 84.2% [17], which is the lowest acceptance rate among all the included studies, to98% [25].

Among studies conducted in Africa, the highest acceptance rate was reported by a Nigerian study (99.3%) [20], with the acceptance rate reported by Nigerian studies being generally above 96.2% [21]. The acceptance rates reported in two studies conducted in Ghana were 94.6% [23] and 94.1% [24]. In Tanzania, a mixed picture was observed, whereby an acceptance rate of 84.2% [17] and 94.3% [18] were reported. Also, an acceptance rate of 96.3% was reported in Sierra Leone [22], and a comparatively much lower rate (88%) was reported in a much older Kenyan study (published in 2014) [16]. A higher acceptance rate (99.3%) was reported by one study conducted in Peru in 2018 [19].

### Factors associated with vaccine acceptability and reasons for hesitancy to accept the malaria vaccine

Overall, four studies reported factors associated with acceptance of RTS,S vaccine [16, 17, 23, 24] while three studies reported reasons for not accepting the vaccine [19, 22, 26]. Of the four reporting factors associated with vaccine acceptance, one study used bivariate analysis [17] while the other three used multivariate analysis to account for confounding factors [16, 23, 24]. Sociodemographic predictors of acceptance reported include residence, tribe age, sex, occupation, and religion. Four studies found that place of residence is associated with vaccine acceptance rate [16, 17, 23, 24]. One study reported that belonging to the age group 27 to 38 years is associated with a higher likelihood of vaccine acceptance [23] while another study reported that caregiver age above 50 years is associated with a lower likelihood of acceptance [16]. One study found that having a female as head of household is associated with a higher likelihood of acceptance of the malaria vaccine [23]. Also, mothers with a child aged 5 to 24 months and those with fewer under-five children were reported to have higher vaccine acceptance [23]. A higher odds of acceptance in Christian caregivers, compared to other religions, was also reported [23]. Also, tribal affiliation has been reported to be associated with the likelihood of vaccine acceptance [17]. Furthermore, having parents who attained a higher level of education is associated with a higher likelihood of vaccine acceptance, relative to those who never received any formal education [24], while having caregivers who never attended school is associated with a lower likelihood of acceptance [16]. Caregivers who are farmershave also been reported to have a higher likelihood of vaccine acceptance [17]. Having poor caregivers was also reported to be a strong predictor of vaccine acceptance [23]. Caregivers’ awareness of a malaria vaccine [23], and their satisfaction with health services [16] were also reported as positive predictors of acceptance. Negative predictors of acceptance included having parents who felt vaccines are too many for children and those whose children developed an abscess as an adverse reaction to childhood vaccines [24].

The reported reasons for not accepting the RTS,S vaccine included vaccine safety [22] and efficacy profile [19, 22], as well as the requirement for receiving multiple doses of the RTS,S injection [19] to attain full immunization. One study reported that respondents believed that a poor level of awareness and lack of vaccine availability would affect acceptability, irrespective of cultural inclination [26].

### Meta-analysis of included studies

**Fig 3:**
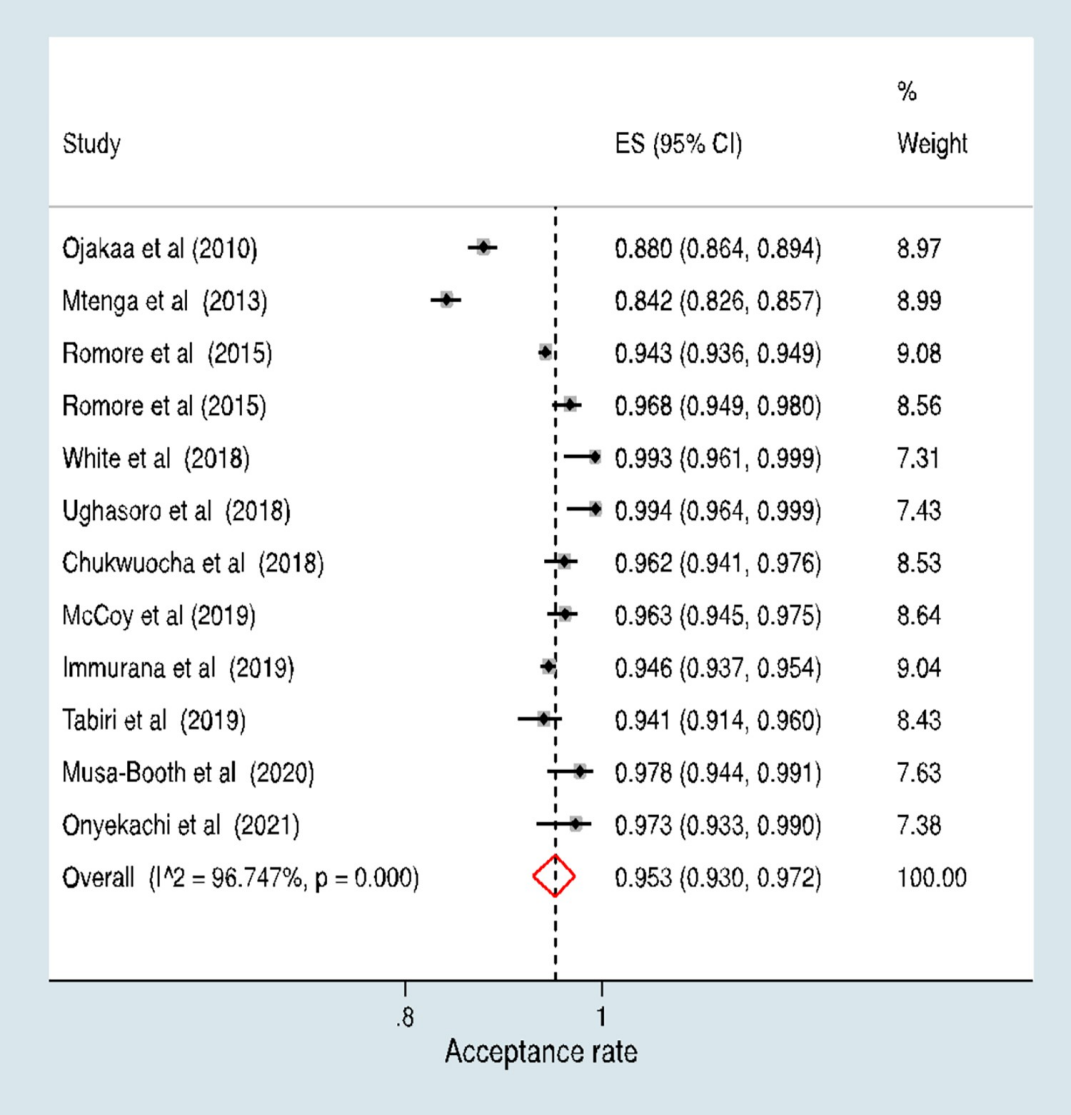
Pooled estimates of malaria vaccine acceptance rates.

**Fig 4:**
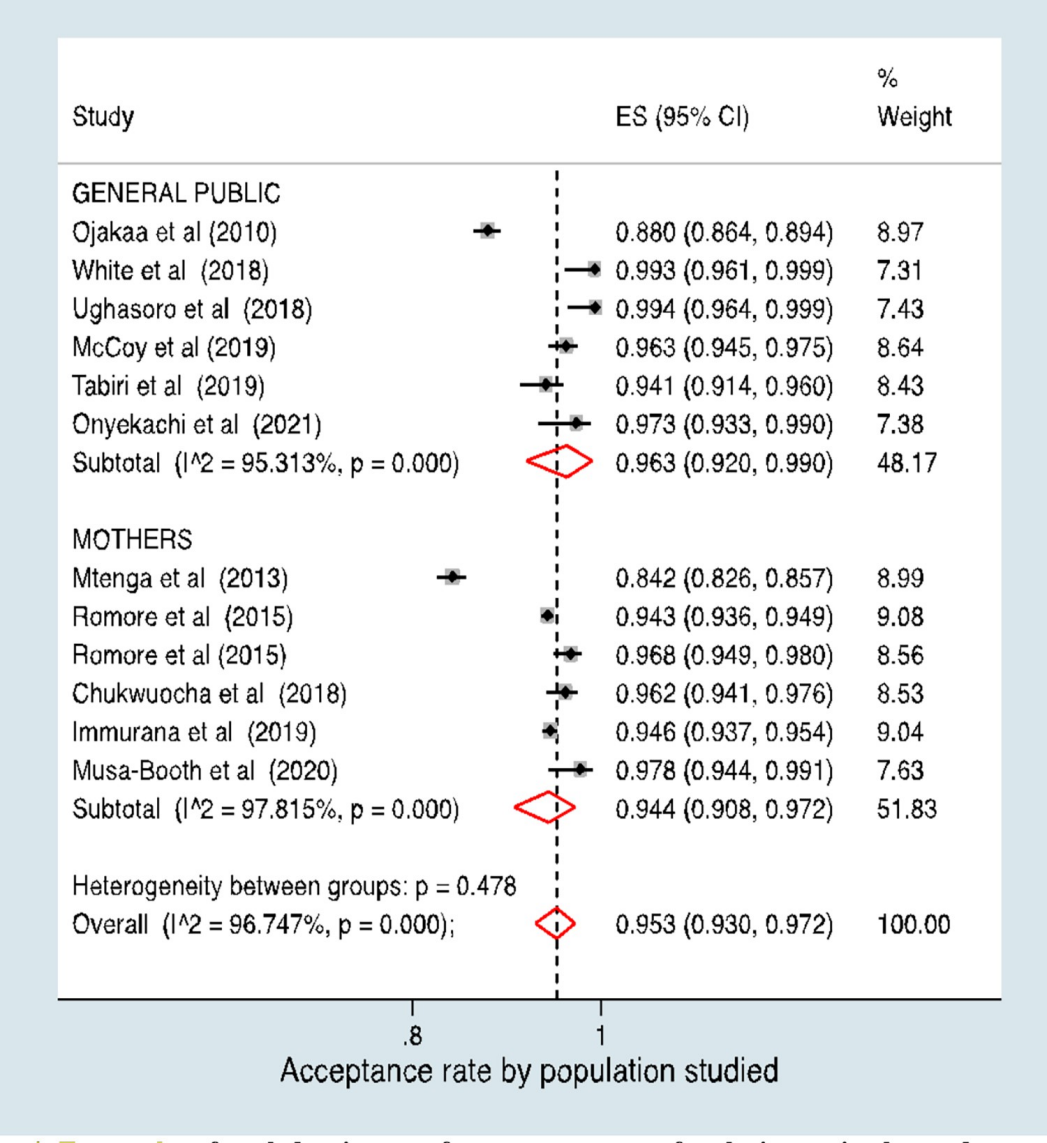
Acceptance of malaria vaccine by study population.

The aggregate malaria vaccine acceptance rate was 95.3% (95% CI, 93.0%–97.2%) **(Fig 3).** However, in a stratified analysis, the pooled acceptance rate for studies conducted among the general population was 96.3% (95% CI, 92.0%–99.0%) and the pooled rate for studies conducted among mothers was 94.4% (95% CI, 90.8%–97.2%) **(Fig 4).** There was no evidence of a difference in the acceptance rates between the two study populations (p = 0.478).

**Fig 5:**
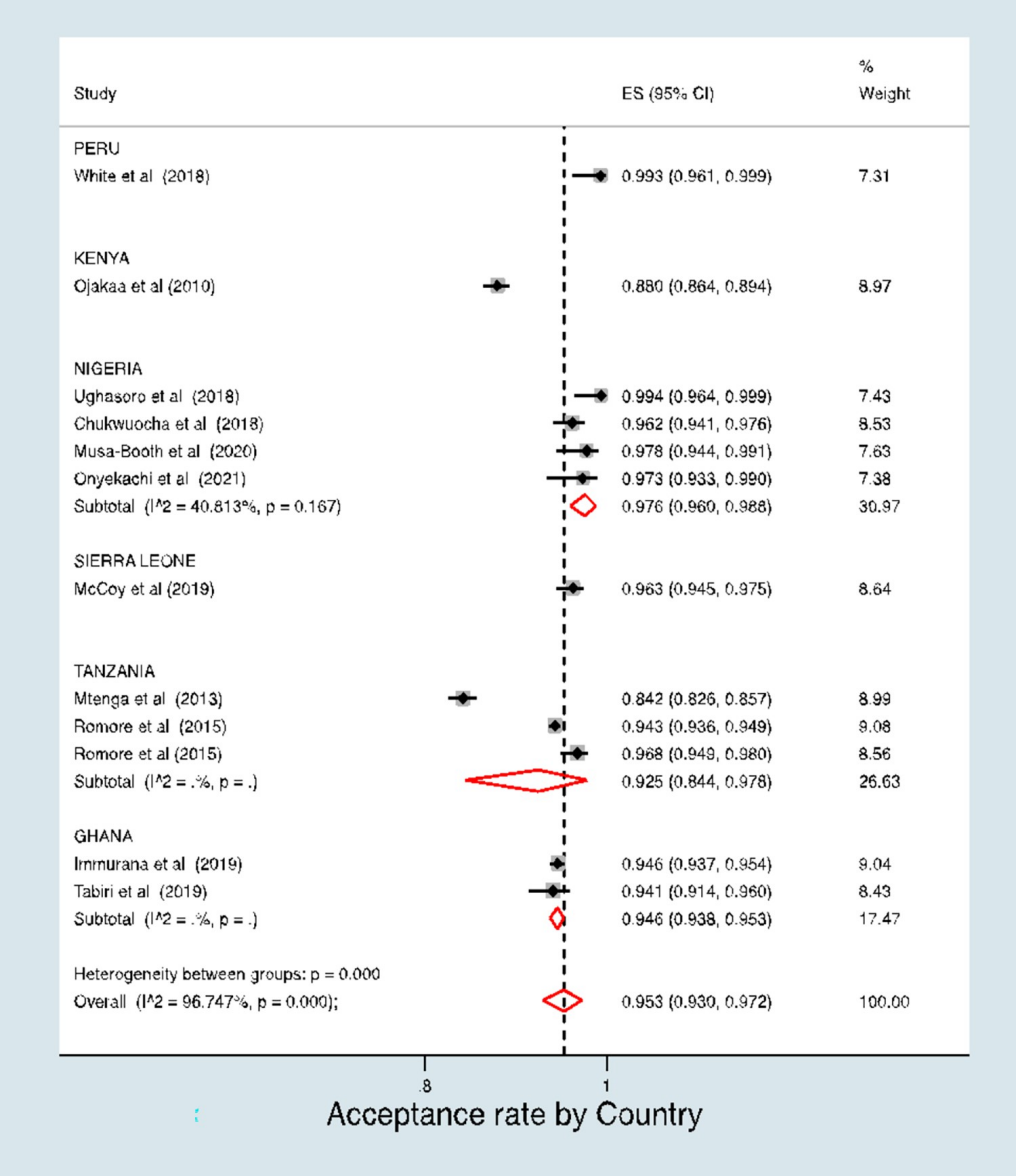
Acceptance malaria vaccine by country of study.

Our analysis demonstrates the existence of a significant degree of heterogeneity in vaccine acceptance rate between countries (p<0.001). Moreover, our stratified analysis (**Fig 5**) shows that acceptance rates varied by country of publication, with the pooled estimate from Nigeria being 97.6% (95% CI: 96.0%-98.8%), the pooled rate from Ghana being 94.6% (95% CI: 93.8%-95.3%), and the rate for studies conducted in Tanzania being 92.5% (95% CI: 84.4%-97.8%).

## Discussion

Following the WHO’s approval of the RTS,S malaria vaccine for under-five children on the 6th of October 2021 [3], we conducted this systematic review and meta-analysis to evaluate the acceptance rates of a malaria vaccine as well as the predictors and reasons for acceptance reported in published literature from LMICs. Our analysis revealed an aggregated random effects pooled prevalence of vaccine acceptance of 95.3% from a sample of 14,666participantsacross 11 studies. Of note, all the reviewed studies were conducted before the WHO approved the RTS,S malaria vaccine on the 6th of October 2021. Also, only two of these studies were published after the onset of the current COVID-19 pandemic—a period that witnessed an unprecedented level of vaccine hesitancy. Therefore, this review highlights the need for new studies, especially in endemic countries of Africa, to re-assess the current attitude of caregivers towards malaria vaccines for children, including factors that may improve actual vaccine uptake in malaria-endemic countries.

The high pooled prevalence of malaria vaccine acceptance observed in this study, relative to a prior systematic review [27], which only reviewed two published studies, is a potentially promising development, given the high incidence of and death rate associated with malaria, particularly among children under five years of age residing in LMICs [3]. Moreover, the burden of malaria in these countries has been reported to have surged to 241 million cases and 627, 000 deaths in 2020, up from 227 million cases and 558, 000 deaths in 2019, largely due to disruptions caused by the COVID-19 pandemic [28]. Importantly, these high acceptance rates, particularly if translated into actual vaccine uptake, may significantly increase the likelihood of attaining the WHO’s target of “reducing global malaria incidence and mortality rates by at least 90% by 2030” [29].

However, it has since been understood that acceptance of a vaccine does not necessarily translate into actual vaccine uptake [30], nor does it imply the absence of hesitancy [31]. This acceptance-uptake gap may even be more pronounced in the context of missed opportunity for vaccination (MOV), defined as “any contact with health services by a child (or adult) who is eligible for vaccination (unvaccinated, partially vaccinated or not up-to-date, and free of contraindications to vaccination), but which does not result in the individual receiving all the vaccine doses for which he or she is eligible” [32]. Given the high prevalence of MOV among children in Africa (27.27%) [33], and LMICs in general (32.2%) [34], there is a pressing need for new studies to identify factors associated with actual uptake of the RTS, S vaccine and factors that may reduce the prevalence of MOV, especially with regards to the RTS, S vaccine.

We identified various sociodemographic factors associated with the acceptance of the malaria vaccine. Similar to previous systematic reviews [35, 36], our analysis indicates that the age of a caregiver is significantly associated with childhood vaccine acceptance. Specifically, we found that mothers aged 27–38 years have higher odds of malaria vaccine acceptance while caregivers aged over 50 years have lower odds of vaccine acceptance [16, 23]. Thus, vaccine campaigns targeting young mothers, especially in communities with poor childhood immunization coverage, may improve the uptake of childhood vaccines. Our review also shows that caregivers who attained some level of education have higher odds of acceptance compared to those who never attended school. This finding echoes the results of previous studies, which showed that attainment of a higher level of education among mothers is a leading predictor of full immunization coverage [4, 37] and that health education interventions targeting caregivers significantly increased parental attitudes toward vaccines [38, 39] and immunization coverage [40, 41]. Therefore, vaccine education campaigns targeting caregivers with a lower level of education may go a long way in improving the acceptance of the RTS,S malaria vaccines among caregivers.

Also, three studies included in this review found that the region of residence of a child’s caregiver is a significant predictor of malaria vaccine acceptance [16, 17, 23]. Variations in socio-demographic characteristics, as well as malaria transmission rates, across regions may potentially explain these regional differences in the RTS,S malaria vaccine acceptance rate. However, further evaluation of the specific factors contributing to these observed regional differences is needed. Furthermore, caregivers who are farmers were reported to have a higher likelihood of vaccine acceptance while richer givers were reported to have a significantly lower likelihood of accepting the vaccine compared to poorer ones. Poor families and households located around farmlands may be more prone to malaria attacks [42, 43]. It is, therefore, reasonable that these particular families may feel more inclined to vaccinate their children against malaria to lessen the frequency of malaria episodes. Additionally, the studies included in this review identified religious denominations of the caregiver as a significant predictor of vaccine acceptance [17, 23], underscoring the potential value of engaging religious authorities in vaccine promotion campaigns. Having a female-headed household is significantly associated with a higher likelihood of malaria vaccine acceptance [23], highlighting the need for vaccine promotion programs to specifically target the chief decision-makers of a family for interventions that have been shown to significantly bolster childhood vaccine uptake, such as sending vaccination reminders to parents [38].

This review also identified the reported reasons for poor vaccine acceptance. One reason common to most reviewed studies is concern about vaccine safety and efficacy, with one study explicitly reporting that parents whose child experienced fever as an adverse effect of immunization are significantly less likely to accept vaccination. Vaccine-related adverse effects have been identified as significant barriers to complete childhood immunization coverage among caregivers [4]. For example, findings from a recently conducted global poll indicated that over half of Nigerians believed that it is definitely or probably true that the harmful effects of vaccines are being deliberately hidden from the public, implying heightened skepticism about transparency among stakeholders and policymakers about vaccine-related information [44]. Given that our study also shows that caregiver’s awareness about a malaria vaccine also increases chances of acceptance, similar to a previous systematic review [45], these findings highlight the need to scale up caregiver education with regards to childhood vaccination in general, and the RTS,S vaccine in particular.

### Limitations

We note some important limitations of our study. Firstly, because all of the studies included in our analysis are cross-sectional, we may not infer the existence of a causal relationship between the exposure and outcome reported in these studies. Secondly, the potential existence of publication bias may limit the validity of our findings. However, stratified analysis of the reviewed studies did not result in notable changes to our overall estimates. Also, except for a single study conducted in Peru, all the studies included in this systematic review were conducted in Africa; thus, the findings of this study may not be generalizable to the global community. Moreover, variability exists in other quality metrics of the included studies.

## Conclusion

In this systematic review and meta-analysis, we found that the acceptance rate of the RTS,S malaria vaccine among children’s caregivers is generally high, but with notable variation across countries. Future efforts should be focused on identifying factors that may improve the actual uptake of the RTS, S vaccine in malaria-endemic communities.

## Supporting information

S1 Fig Funnel plot of the included studies to estimate pooled prevalence of malaria vaccine acceptance rate among the residents of LMIC. (TIF)

S1 Table Literature search strategy. (DOCX)

S2 Table Quality assessment scoring. (DOCX)

S3 Table PRISMA 2009 checklist. (DOC)

## List of abbreviations and acronyms

CS: Cross-sectional Study
IRIS: Institutional Repository for Information Sharing
JBI: Joanna Briggs Institute
LMICs: Low- and middle-income countries
MeSH: Medical Subject Headings
MOV: Missed Opportunity for Vaccination
NOS: Newcastle-Ottawa scale
NR: Not Reported
PRISMA: Preferred Reporting Item for Systematic Review and Meta-analysis
PROSPERO: International Prospective Register for Systematic Review
QC: Qualitative Comments
TBV: Transmission-Blocking Vaccine
WHO: World Health Organization
