# Supplementary figures and images for: A systematic review and meta-analysis of the prevalence of caregiver acceptance of malaria vaccine for under-five children in low-income and middle-income countries (LMICs)
Source: PLoS One. 2022 Dec 1;17(12):e0278224. doi: 10.1371/journal.pone.0278224 (PMC9715017; doi:10.1371/journal.pone.0278224)

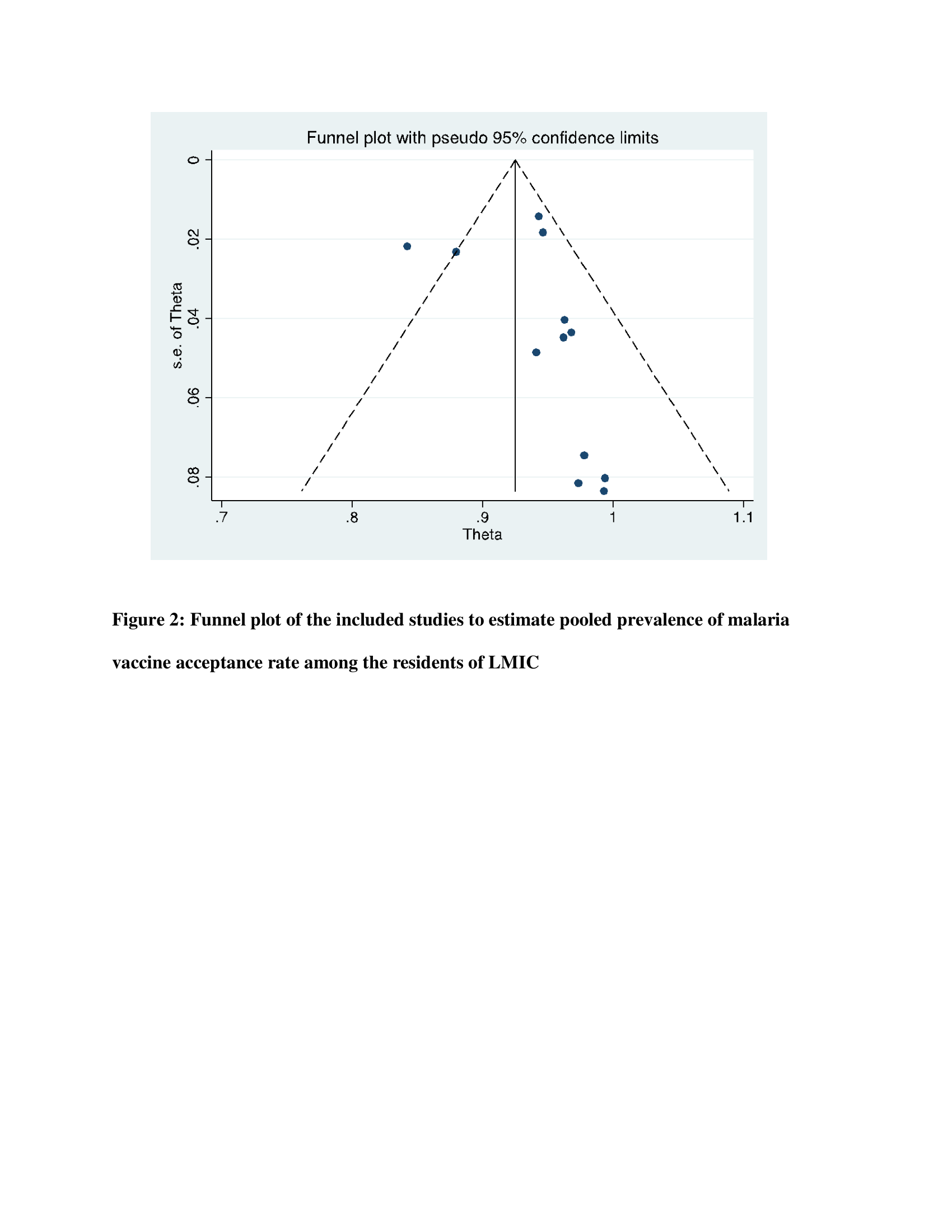

Supplement: S1 Fig — (TIF) [file pone.0278224.s001.tif]
