# Supplementary material for: A systematic review and meta-analysis of the prevalence of caregiver acceptance of malaria vaccine for under-five children in low-income and middle-income countries (LMICs)
Source: PLoS One. 2022 Dec 1;17(12):e0278224. doi: 10.1371/journal.pone.0278224 (PMC9715017; doi:10.1371/journal.pone.0278224)
Supplement: S1 Table — (DOCX) [file pone.0278224.s002.docx]

**Appendix 1: Search strategy**

**PUBMED**

| 1 | "Malaria"[Mesh] OR OR OR OR OR OR OR |
| --- | --- |
| 2 | “vaccine”[Title/Abstract] OR “vaccination”[Title/Abstract] OR “immunization” [Title/Abstract] |
| 3 | 1 OR 2 |
| 4 | “acceptance” [Title/Abstract] OR “uptake”[Title/Abstract] OR “willingness”[Title/Abstract] OR “awareness”[Title/Abstract] OR “perception” [Title/Abstract] |
| 5 | "Developing Countries"[Mesh] OR "Africa"[Mesh] OR "Asia"[Mesh] OR "Caribbean Region"[Mesh] OR "Central America"[Mesh] OR "South America"[Mesh] OR "Europe, Eastern"[Mesh] OR "Middle East"[Mesh] |
| 6 | LMIC[Title/Abstract] OR LIC[Title/Abstract] OR MIC[Title/Abstract] OR "third world"[Title/Abstract] OR (((“low and middle income” OR “low- and middle- income” OR LAMI OR “resource limited” OR “resource-limited” OR "low income" OR "low-income" OR “middle-income” OR "less developed" OR "middle income" OR "underdeveloped" OR "developing") AND (countr* OR nation* OR setting*))[Title/Abstract]) OR Afghanistan*[Title/Abstract] OR Benin*[Title/Abstract] OR "Burkina Faso"[Title/Abstract] OR Burundi*[Title/Abstract] OR "Central African Republic"[Title/Abstract] OR Chad*[Title/Abstract] OR Comoros*[Title/Abstract] OR "Democratic Republic of the Congo"[Title/Abstract] OR Eritrea*[Title/Abstract] OR Ethiopia*[Title/Abstract] OR Gambia*[Title/Abstract] OR Guinea*[Title/Abstract] OR Guinea-Bissau*[Title/Abstract] OR Haiti*[Title/Abstract] OR Korea*[Title/Abstract] OR Liberia*[Title/Abstract] OR Madagascar*[Title/Abstract] OR Malawi*[Title/Abstract] OR Mali*[Title/Abstract] OR Mozambique*[Title/Abstract] OR Nepal*[Title/Abstract] OR Niger*[Title/Abstract] OR Rwanda*[Title/Abstract] OR Senegal*[Title/Abstract] OR "Sierra Leone"[Title/Abstract] OR Somalia*[Title/Abstract] OR "South Sudan"[Title/Abstract] OR Tanzania*[Title/Abstract] OR Togo*[Title/Abstract] OR Uganda*[Title/Abstract] OR Zimbabwe*[Title/Abstract] OR Armenia*[Title/Abstract] OR Bangladesh*[Title/Abstract] OR Bhutan*[Title/Abstract] OR Bolivia*[Title/Abstract] OR "Cabo Verde"[Title/Abstract] OR Cambodia*[Title/Abstract] OR Cameroon*[Title/Abstract] OR Congo*[Title/Abstract] OR "Côte d'Ivoire"[Title/Abstract] OR Djibouti*[Title/Abstract] OR Egypt*[Title/Abstract] OR "El Salvador"[Title/Abstract] OR Ghana*[Title/Abstract] OR Guatemala*[Title/Abstract] OR Honduras*[Title/Abstract] OR India*[Title/Abstract] OR Indonesia*[Title/Abstract] OR Kenya*[Title/Abstract] OR Kiribati*[Title/Abstract] OR Kosovo*[Title/Abstract] OR Kyrgyz*[Title/Abstract] OR "Lao PDR"[Title/Abstract] OR Lesotho*[Title/Abstract] OR Mauritania*[Title/Abstract] OR Micronesia*[Title/Abstract] OR Moldova*[Title/Abstract] OR Mongolia*[Title/Abstract] OR Morocco*[Title/Abstract] OR Myanmar*[Title/Abstract] OR Nicaragua*[Title/Abstract] OR Nigeria*[Title/Abstract] OR Pakistan*[Title/Abstract] OR "Papua New Guinea"[Title/Abstract] OR Philippines*[Title/Abstract] OR Samoa*[Title/Abstract] OR "São Tomé"[Title/Abstract] OR "Solomon Islands"[Title/Abstract] OR "Sri Lanka"[Title/Abstract] OR Sudan*[Title/Abstract] OR Swaziland*[Title/Abstract] OR Syria*[Title/Abstract] OR Tajikistan*[Title/Abstract] OR Timor-Leste*[Title/Abstract] OR Tonga*[Title/Abstract] OR Tunisia*[Title/Abstract] OR Ukraine*[Title/Abstract] OR Uzbekistan*[Title/Abstract] OR Vanuatu*[Title/Abstract] OR Vietnam*[Title/Abstract] OR "West Bank"[Title/Abstract] OR Gaza*[Title/Abstract] OR Yemen*[Title/Abstract] OR Zambia*[Title/Abstract] OR Albania*[Title/Abstract] OR Algeria*[Title/Abstract] OR Samoa*[Title/Abstract] OR Angola*[Title/Abstract] OR Azerbaijan*[Title/Abstract] OR Belarus*[Title/Abstract] OR Belize*[Title/Abstract] OR Bosnia*[Title/Abstract] OR Botswana*[Title/Abstract] OR Brazil*[Title/Abstract] OR Bulgaria*[Title/Abstract] OR China*[Title/Abstract] OR Colombia*[Title/Abstract] OR "Costa Rica"[Title/Abstract] OR Cuba*[Title/Abstract] OR Dominica*[Title/Abstract] OR "Dominican Republic"[Title/Abstract] OR Ecuador*[Title/Abstract] OR "Equatorial Guinea"[Title/Abstract] OR Fiji*[Title/Abstract] OR Gabon*[Title/Abstract] OR Georgia*[Title/Abstract] OR Grenada*[Title/Abstract] OR Guyana*[Title/Abstract] OR Iran*[Title/Abstract] OR Iraq*[Title/Abstract] OR Jamaica*[Title/Abstract] OR Jordan*[Title/Abstract] OR Kazakhstan*[Title/Abstract] OR Lebanon*[Title/Abstract] OR Libya*[Title/Abstract] OR Macedonia*[Title/Abstract] OR Malaysia*[Title/Abstract] OR Maldives*[Title/Abstract] OR "Marshall Islands"[Title/Abstract] OR Mauritius*[Title/Abstract] OR Mexico*[Title/Abstract] OR Montenegro*[Title/Abstract] OR Namibia*[Title/Abstract] OR Palau*[Title/Abstract] OR Panama*[Title/Abstract] OR Paraguay*[Title/Abstract] OR Peru*[Title/Abstract] OR Romania*[Title/Abstract] OR Russia*[Title/Abstract] OR Serbia*[Title/Abstract] OR "South Africa"[Title/Abstract] OR "St. Lucia"[Title/Abstract] OR "St. Vincent and the Grenadines"[Title/Abstract] OR Suriname*[Title/Abstract] OR Thailand*[Title/Abstract] OR Turkey*[Title/Abstract] OR Turkmenistan*[Title/Abstract] OR Tuvalu*[Title/Abstract] OR Venezuela*[Title/Abstract] |
| 7 | 6 OR 7 |
| 8 | 3 AND 4 AND 7 |
