# Supplementary material for: A systematic review and meta-analysis of the prevalence of caregiver acceptance of malaria vaccine for under-five children in low-income and middle-income countries (LMICs)
Source: PLoS One. 2022 Dec 1;17(12):e0278224. doi: 10.1371/journal.pone.0278224 (PMC9715017; doi:10.1371/journal.pone.0278224)
Supplement: S2 Table — (DOCX) [file pone.0278224.s003.docx]

**Quality Assessment Scoring**

**Table 1 Results of the consensus critical appraisal of the included studies**

| Author (first) | Study design |  | Selection |  |  | Comparability | Outcome |  |  |  |
| --- | --- | --- | --- | --- | --- | --- | --- | --- | --- | --- |
|  |  | Representativeness | Sample size | Non-respondents | Ascertainment of exposure | Based on design | Assessment of outcome | Statistical test | Total score | Quality grade |
|  |  | of the sample |  |  |  | and analysis |  |  |  |  |
| Ojakaa et al. | Cross- sectional | * | * |  | * | * | ** | * | 7 | Moderate |
| Mtenga et al | Cross- sectional | * | * | * | * | * | * | * | 7 | Moderate |
| Romore et al | Cross- sectional | * | * | * | * | ** | * | * | 8 | High |
| Romore et al | Cross- sectional | * | * | * | * | ** | * | * | 8 | High |
| White et al | Cross- sectional | * | * | * | * | * | * | * | 7 | Moderate |
| Ughasoro et al | Cross- sectional | * |  |  | * |  | * |  | 3 | Low |
| Chukwuocha et al | Cross- sectional | * | * |  | ** |  | * | * | 6 | Moderate |
| McCoy et al | Cross- sectional | * | * | * | * |  | * |  | 5 | Moderate |
| Immurana et al | Cross- sectional | * | * | * | * | ** | ** | * | 9 | High |
| Tabiri et al | Cross- sectional | * | * |  | * | ** | ** | * | 8 | High |
| Musa-Booth et al | Cross- sectional | * | * |  | * | ** | ** | * | 8 | High |
| Onyekachi et al | Cross- sectional | * | * | * |  |  | * |  | 4 | Low |
